# Nearly Complete Genome Sequences of Two Bluetongue Viruses Isolated during the 2020 Outbreak in the Grand Duchy of Luxembourg

**DOI:** 10.1128/MRA.00210-21

**Published:** 2021-04-08

**Authors:** Frank Vandenbussche, Manon Bourg, Elisabeth Mathijs, David J. Lefebvre, Ilse De Leeuw, Andy Haegeman, Laetitia Aerts, Steven Van Borm, Kris De Clercq

**Affiliations:** a Sciensano, Scientific Directorate Animal Infectious Diseases, Ukkel, Belgium; b Sciensano, Exotic Viruses and Particular Diseases Unit, Ukkel, Belgium; c Laboratoire de Médecine Vétérinaire de l’État, Veterinary Services Administration, Dudelange, Grand Duchy of Luxembourg; KU Leuven

## Abstract

Bluetongue is one of the major diseases of ruminants listed by the World Organisation for Animal Health. Bluetongue virus serotype 8 (BTV-8) has been considered enzootic in France since 2018. Here, we report the nearly complete genome sequences of two BTV-8 isolates from the 2020 outbreak in the Grand Duchy of Luxembourg.

## ANNOUNCEMENT

Bluetongue is a vector-borne disease affecting both domestic and wild ruminants. The disease is caused by the *Bluetongue virus* (BTV), which is the type species of the genus *Orbivirus*, within the family *Reoviridae* (1, 2). In August 2006, BTV serotype 8 (BTV-8) emerged for the first time in northwestern Europe and rapidly spread across large parts of the continent (3). The outbreak was successfully controlled through a large-scale vaccination campaign, with only a small number of cases reported in Europe in 2010 (4). However, in 2015, BTV-8 reemerged in France (5), from where it spread to neighboring countries, including Switzerland, Germany, and Belgium.

In 2020, BTV reemerged in the Grand Duchy (GD) of Luxembourg, with 25 outbreaks being detected in cattle all across the country. To gain more insights into the origin of this reemergence, we isolated and sequenced BTV from the blood of cattle collected during the first two outbreaks. The viruses were isolated in embryonated chicken eggs and subsequently passaged twice on BHK-21 cells (6). Total RNA was extracted from the cell pellet of an infected 175-cm^2^ monolayer showing 100% cytopathic effect using a NucleoSpin RNA virus kit (Macherey-Nagel) and treated with Baseline-ZERO DNase (Lucigen) and mung bean nuclease (New England Biolabs). cDNA was synthesized with SuperScript IV reverse transcriptase (Thermo Fisher Scientific) using a mixture of random hexamers and primers targeting the conserved BTV termini (7). Nextera XT sequencing libraries were prepared from 1 isolate per outbreak and analyzed on a MiSeq system using a MiSeq reagent kit v3 (2 × 300 bp; Illumina), yielding approximately 2.5 million read pairs per sample. After removal of adapter sequences and low-quality bases with Trimmomatic v0.38 (8), both data sets were enriched for BTV using mirabait with a target data set containing 2,517 BTV sequences from GenBank (9). *De novo* assembly of the segments was performed using IVA v1.0.8 (10), MIRA v5rc1 (9), and SPAdes v3.9.0 (11). The resulting contigs of the different assemblers were combined into single consensus sequences. Nucleotide variants were called using the GATK v4.1.3.0 best practices pipeline (12, 13). Finally, the sequences were annotated with GATU (14), using BTV isolate FRA2017-7300 (GenBank accession numbers MN837938, MN838139, MN838307, MN838410, MN838571, MN838719, MN838862, MN839113, MN839158, and MN839405) as the reference genome sequence.

Although only 14 to 39% of the total reads were derived from BTV, we were able to reconstruct complete genome sequences with a high depth of coverage for both data sets (Table 1). Comparison of the genome segments revealed that the isolates are closely related with identities at the nucleotide level ranging from 99.82 to 100.00%. We also compared the genome segments with other BTV segments from publicly available databases. BLAST analysis showed that all the segments are closely related to recent BTV-8 isolates from France, with BTV isolate FRA2017-7300 displaying the highest percent identity (BTV8/LUX/2020/1, 99.80 to 100.00%; BTV8/LUX/2020/2, 99.73 to 100.00%). As expected, we observed the highest number of differences in segment 2, which is known to be the most variable segment (Table 1). The whole-genome sequence data thus strongly suggest that both GD Luxembourg isolates are derived from recent French outbreaks.

**TABLE 1:** Summary of the assembly results of the BTV8/LUX/2020/1 and BTV8/LUX/2020/2 data sets and comparison with BTV isolate FRA2017-7300

Isolate	Segment	Size (bp)	GC content (%)	Depth of coverage (×)	SNPs^*a*^ (position/substitution)	GenBank accession no.
BTV8/LUX/2020/1	1	3,944	42.22	1,553	**134/A→R** 3566/G→A	MW528437
	2	2,939	42.67	1,662	281/C→T 2051/T→C 2663/A→G **2901/T→K**	MW528438
	3	2,772	43.58	1,826	**1777/C→T** 2327/C→T 2627/G→A	MW528439
	4	1,981	43.61	1,994	**354/G→R** **1278/A→G** 1511/G→C 1625/G→A	MW528440
	5	1,776	45.50	1,832	**879/G→A**	MW528441
	6	1,637	44.04	2,447	26/A→G 480/C→A 729/A→G	MW528442
	7	1,156	46.54	1,850	746/C→T **1061/T→W**	MW528443
	8	1,125	45.24	2,584	MW528444
	9	843	49.67	2,385	843/T→C	MW528445
	10	822	45.86	1,173	MW528446
BTV8/LUX/2020/2	1	3,944	42.24	14,223	1571/A→G 3566/G→A	MW528447
	2	2,939	42.57	15,327	281/C→T **688/A→R** **1540/G→A** **1783/C→T** **1948/C→T** 2051/T→C 2663/A→G **2901/T→K**	MW528448
	3	2,772	43.54	17,384	**1777/C→T** **2086/C→Y** 2327/C→T 2627/G→A	MW528449
	4	1,981	43.66	18,194	**1278/A→G** 1511/G→C 1625/G→A	MW528450
	5	1,776	45.55	16,824	MW528451
	6	1,637	43.98	22,451	480/C→A 729/A→G 1455/T→Y	MW528452
	7	1,156	46.54	17,146	746/C→T	MW528453
	8	1,125	45.24	22,406	58/T→C **623/G→A**	MW528454
	9	1,049	49.67	22,006	843/T→C	MW528455
	10	822	45.86	10,389	MW528456

a Nonsynonymous substitutions are shown in bold. SNPs, single nucleotide polymorphisms.

### Data availability.

The complete genome sequences have been deposited in GenBank under accession numbers MW528437 through MW528446 (BTV8/LUX/2020/1) and MW528447 through MW528456 (BTV8/LUX/2020/2), and the raw sequencing reads are available in the SRA database under BioProject accession number PRJNA705052.
